# Spatiotemporal Distribution of Host Plants of Dusky Cotton Bug, *Oxycarenus laetus,* Kirby 1891 at Different Climatic Zones of Sindh, Pakistan

**DOI:** 10.3390/insects15110889

**Published:** 2024-11-14

**Authors:** Muhammad Mithal Rind, Hakim Ali Sahito, Gregorio Vono

**Affiliations:** 1 Department of Zoology, Shah Abdul Latif University, Khairpur 66111, Pakistan; 2 Istituto di Istruzione Superiore “V. Emanuele II–B. Chimirri” Catanzaro, Via V. Cortese,1, 88100 Catanzaro, Italy

**Keywords:** ecology, insect-hosts interactions, distribution patterns, climate influence, agricultural pests, species distribution

## Abstract

The study aimed to identify the host plants of the Dusky Cotton Bug (*Oxycarenus laetus*), in various agro-ecological zones of Sindh, Pakistan, 2019. Samples were collected bi-weekly within 20 km of the Cotton Agriculture Research Station in each district of Sindh. The pest population is categorized into three levels: below 25; 25 to 49 and 50 or more adults and nymphs. The study identified approximately 63 host plants across 31 families. The highest overall mean of pest populations was recorded on Ladyfinger (Okra), with 51.75 ± 8.15 bugs per shoot at Kotdiji and 53.71 ± 4.68 per shoot at Sakrand, both in the Malvaceae family. A high overall mean of populations was also observed on Mango (Anacardiaceae) with 51.65 ± 11.99 bugs per shoot at Kotdiji and 46.42 ± 5.84 per shoot at Sakrand on Orange (Rutaceae) with 42.07 ± 8.93 bugs per shoot at Kotdiji and 45.17 ± 4.11 per shoot at Sakrand, and on Eucalyptus and Guava (Myrtaceae) with 29.75 ± 6.76 per shoot at Kotdiji and 26.53 ± 3.71 per shoot at Tandojam, respectively. Additionally, the pest was found on Jujube (Rhamnaceae) with an overall mean population of 26.92 ± 3.52 per shoot at Sakrand The results indicate that the Dusky cotton Bug is most active at the end of summer and the beginning of winter, preferring high-opened cotton bolls during period of slightly lower temperatures and humidity for overwintering from December to March on seed-producing host plants. These findings are crucial for understanding the host plant preferences of the Dusky Cotton Bug, and for implementing effective Integrated Pest Management (IPM) strategies.

## Simple Summary

This study aimed to identify the host plants of the Dusky Cotton Bug *Oxycarenus laetus*, a polyphagous pest, in various agro-ecological zones of Sindh, Pakistan in 2019. Samples were collected bi-weekly within a 20 km radius of the Cotton Agriculture Research Station in each district. The pest population was categorized into three levels: below 25, 25 to 49, and 50 or more adults and nymphs. The study identified about 63 host plants across 31 families. The highest pest populations were found on Okra, Mango, Orange, Eucalyptus, Guava, and Jujube. The Dusky Cotton Bug is most active at the end of summer and the beginning of winter, preferring high, opened cotton bolls during cooler, slightly humid conditions for overwintering. These findings are important for understanding the host plant preferences of the Dusky Cotton Bug and for developing effective Integrated Pest Management (IPM) strategies.

## 1. Introduction

Cotton cultivation is crucial to Pakistan’s economy, positioning the country as one of the leading cotton producers in the world. This crop not only serves as a primary raw material for the textile industry but also generates millions of jobs, thereby supporting rural livelihoods. Moreover, cotton farming is a significant source of income for farmers, contributing to food security and economic development in various regions. The sector faces challenges such as climate change and pest infestations, highlighting the need for sustainable agricultural practices and innovative approaches to enhance productivity [1].

The Dusky Cotton Bug, *Oxycarenus laetus* Kirby (Hemiptera: Lygaeidae), is commonly famous as a seed bug that poses a significant threat to cotton crops, particularly in Pakistan [2,3]. This pest is a newly emerging menace to the cotton industry [4] and has a global distribution [5], including islands near the mainland and ports of the USA. Peak populations have been observed in Florida [6], where both adults and nymphs feed on immature and mature seeds, causing them to remain unripe and lightweight [7]. Additionally, the Dusky Cotton Bug extracts cell sap from the reproductive parts of plants, degrading lint quality and impeding the efficient ginning of cotton fibers.

The large infestations of this pest on cotton squares cause the color to change from green to pale yellow eventually; causing them to shed [8]. This pest significantly diminishes both the quality and quantity of the seed [9,10], and, if left uncontrolled, it can lead to substantial losses in cotton fiber [11]. Infestation results in reduced seed germination, lower oil content, and lint discoloration during ginning [12,13]. The insect damages the seed cotton embryo, decreasing seed viability [14,15]. The highest population of DCB is observed from October to November [16], attributed to breeding on highly opened bolls of cotton crops at slightly lower temperatures. Both adults and nymphs feed on seeds and sometimes on leaves and young stems, leading to a 6–6.8% reduction in cotton yield, seed weight, and oil contents [17]. DCB completes six to seven generations annually [18].

The Dusky Cotton Bug persists throughout the year on various alternate host plants such as Okra, Cotton, Lemon, Guava, and Moringa [11,19]. In Pakistan, it is found year-round on Mango, Abutilon, China Rose, Phalsa, Sweet lemon, Barseem, Wheat, Ficus, Jasmine, Date palm, Chilies, Cotton [20], Eucalyptus, and Neem [21] with numerous alternate host plants [22]. Currently, there has been no identification of various alternative host plants or research conducted in the different agro-ecological zones of Sindh. Therefore, this study aims to address this gap by investigating the pest populations and their interactions with alternative host plants in these regions. The bug feeds on young petiole tissues [23], and seeds produced by alternate host plants at different intervals throughout the year serve as a primary food source [24]. Occasionally, the last generation is overwintered on weed branches, leaves, grasses, or other such locations [18]. As a polyphagous and migratory insect, it predominantly feeds on plants from the Malvaceae family [23,25]. Sucking complexes on cotton and various host plants such as *Phenacoccus. solenopsis* are reported on plants like Fabaceae, Solanaceae, Euphorbiaceae, Asteraceae, and Malvaceae [26].

Weather factors significantly influenced the DCB population [22,24], with the population peaking from September to November, reaching its zenith in October [27]. In different agro-ecological zones of Sindh, the pest appears on cotton in early September, particularly in Kotdiji, an agro-eco zone of Sindh. The climatic conditions, such as temperatures ranging from 34 to 27 °C and relative humidity between 28 and 21%, are ideal for pest multiplication [28]. The geographical dispersal, abundance, and infestation of these insects are directly linked to their feeding and reproductive capabilities on multiple hosts and their environmental adaptability [29]. Considering the high pest population on cotton and other host plants, the research aims to identify the host plants of the Dusky Cotton Bug in areas surrounding Cotton Agriculture Research Stations located in various agro-ecological zones of Sindh, Pakistan. This study aims to provide valuable insights into the various host plants associated with the Dusky Cotton Bug, thereby benefiting the farming community, emerging researchers, agricultural extension workers, and all stakeholders involved in cotton management. By enhancing the understanding of the pest’s host plants, the findings will contribute to more effective pest management strategies. Additionally, the conclusions regarding abiotic factors will offer guidance for implementing preventive measures to mitigate pest migration to cotton crops across the diverse agro-ecological zones of Sindh.

## 2. Materials and Methods

### 2.1. Experimental Field Area

This study aimed to evaluate the alternate host plants of Dusky Cotton Bug across various agro-ecological zones in the Sindh province of Pakistan in 2019. The study included four treatments: T1 (District: Hyderabad), T2 (Shaheed Benazirabad), T3 (Khairpur), and T4 (Ghotki), with each treatment replicated at bi-weekly (fortnightly) intervals. Additionally, the study focused on monitoring the spatiotemporal distribution of the pest across various agro-ecological zones. Experimental fields and their surrounding vicinities at Cotton Agriculture Research Stations were selected in four districts of Sindh: Hyderabad (Tandojam), Shaheed Benazirabad (Sakrand), Khairpur (Kotdiji), and Ghotki (Sarhad). Data were collected fortnightly from different crops, vegetables, fruits, herbs, shrubs, trees, ornamental plants, medicinal plants, and weeds within a 20 km radius of each Cotton Agriculture Research Station, as described by [30]. The study period spanned from 1 January to 31 December 2019.

### 2.2. Sampling Method

Sampling of the Dusky Cotton Bug (DCB) was categorized into three levels:

A. Less than 25 adults and nymphs per sample (10.0 cm).
B. 25 to 49 adults and nymphs per sample (10.0 cm).
C. 50 or more adults and nymphs per sample (10.0 cm), as described by [20].

Random sampling was conducted on buds, flowers, leaves, and inflorescences of host plants, including gardening trees. Over ten samples per host plant were examined, including pests found under leaf sheaths. The DCB was collected using a camel hairbrush into the plastic jars and carefully counted as reported by [20]. Meteorological data (temperature °C and relative humidity %) were recorded using the Accu Weather Mobile 3.9, software.

### 2.3. Statistical Analysis

The pest population data on different host plants were statistically analyzed using analysis of variance (ANOVA), and means were separated by the least significant differences (LSD) test at Alpha = 0.05. Graphs were prepared through Graph Pad Prism 6 software.

## 3. Results

### 3.1. Identification of Plant Families

The results of this study, as presented in Table 1, indicate that the Dusky Cotton Bug (*Oxycarenus laetus*) infests sixty-three alternate host plants across thirty-one families at various locations in Sindh, Pakistan. The various identified host plants are presented in abbreviated form as follows: T = Tree; PC = Pulse crops; TP = Tobacco plant; OP = Ornamental plant; S = Shrubs; FC = Fodder crops; V = Vegetable; OSC = Oilseed crop; CC = Cereal crop; W = Weed; SP = Salad plant; MP = Medicinal plant; SC = Sugar crop; FP = Fruit plant. The identified host plants included a diverse range of plant types: cereal crops (n = 4), oilseed crops (n = 4), fodder crops (n = 3), sugar crops (n = 1), medicinal crops (n = 1), tobacco plants (n = 1), pulse (n = 5), vegetables (n = 15), salad plants (n = 1) ornamental plants (n = 3), fruit trees (n = 10), fruit plants (n = 1), trees (n = 11), shrubs (n = 5), and weeds (n = 2).

Fortnightly, pest population data for each affected plant family were statistically analyzed using analysis of variance (ANOVA). The results indicated significant differences in pest populations among certain plant families, with the following F and *p* values:

Myrtaceae: F_(6.53)_; (Df = 3, 7); *p* = 0.0072

Moraceae: F_(2.14)_; (Df = 3, 7); *p* = 0.1480

Rutaceae: F_(17.03);_ (Df = 3, 4); *p* = 0.0150

Fabaceae: F_(18.07)_; (Df = 3, 15); *p* = 0.0030

Gramineae: F_(2.92)_; (Df = 3, 15); *p* = 0.0770

Cucurbitaceae: F_(1.59)_; (Df = 3, 12); *p* = 0.2700

Malvaceae: F_(4.47)_; (Df = 3, 4); *p* = 0.1020

Brassicaceae: F_(27.19)_; (Df = 3, 7); *p* = 0.0090

Crucifereae: F_(4.86)_; (Df = 3, 7); *p* = 0.0560

Apiaceae: F_(4.22)_; (Df = 3, 4); *p* = 0.1090

Poaceae: F_(1.53)_; (Df = 3, 4); *p* = 0.2840

Asteraceae: F_(36.64)_; (Df = 3, 4); *p* = 0.0030

Papilionaceae: F_(32.58)_; (Df = 3, 4); *p* = 0.0050

The overall mean of pests in different agro-ecological zones of Sindh highlighted the highest pest populations were recorded on alternate host plants from the following families:

Anacardiaceae: *Mangifera indica* L. (43.95 ± 3.74)

Malvaceae: *Abelmoschus esculentus* L. (43.18 ± 5.54), *Hibiscus* sp., (25.38 ± 2.06)

Rutaceae: *Citrus sinensis* Osbeck (39.91 ± 2.25), *Citrus aurantifolia* (21.60 ± 2.09)

Rhamnaceae: *Zizyphus mauritiana* Lamk (21.89 ± 2.39),

Myrtaceae: *Eucalyptus camaldulensis* Dehnh (22.55 ± 3.53);

*Psidium guajava* L. (17.79 ± 3.92), Palmaceae: *Phoenix dactylifera* L. (14.03 ± 1.07),

The high pest population on these alternate host plants is attributed to the harvesting of the primary host, the cotton crop, which leads to reduced availability of food in the vicinity and drives pest migration. Seasonal changes, particularly from mid-October to March, also influence pest migration, with climate factors during the summer favoring migration from alternate host plants to cotton across different agro-ecological zones of Sindh.

### 3.2. Maximum and Minimum Pest Population

**Figure 1:**
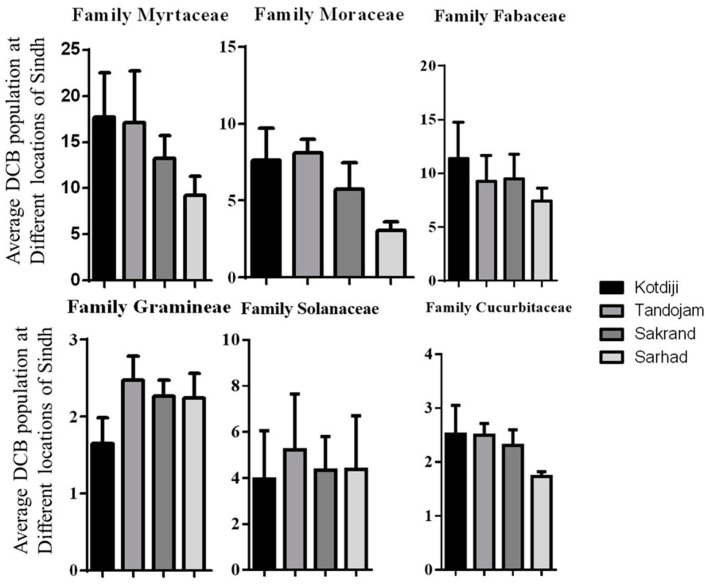
The overall mean of DCB on host plants of different plant families in diverse climatic zones of Sindh.

The current research (Figure 1) indicated that the highest pest populations were recorded in Kotdiji (17.69 DCB/shoot), Tandojam (17.09 DCB/shoot), Sakrand (13.25 DCB/shoot), and Sarhad (9.20 DCB/shoot) on plants from the Myrtaceae family. Similarly, high DCB populations were observed in Tandojam (8.12 DCB/shoot) and Kotdiji (7.63 DCB/shoot) on plants from the Moraceae family. In the Fabaceae family, pest numbers were highest in Kotdiji (11.37 DCB/shoot) and Sakrand (9.46 DCB/shoot). Conversely, lower numbers of Dusky Cotton Bug were observed in Tandojam (2.48 DCB/shoot) and Sakrand (2.26 DCB/shoot) on plants from the Gramineae family. Additionally, DCB populations were noted in Kotdiji (2.52 DCB/shoot) and Tandojam (2.50 DCB/shoot) on plants from the Cucurbitaceae family. A statistical analysis of the plant families was conducted using analysis of variance (ANOVA). The results indicated non-significant differences in DCB populations among the plant families, with the following values: Myrtaceae (F = 0.32; Df = 3, 7; *p* = 0.81), Moraceae (F = 1.38; Df = 3, 7; *p* = 0.32), Fabaceae (F = 0.43; Df = 3, 15; *p* = 0.73), Gramineae (F = 1.42; Df = 3, 15; *p* = 0.27), Solanaceae (F = 0.07; Df = 3, 15; *p* = 0.97), and Cucurbitaceae (F = 1.30; Df = 3, 12; *p* = 0.31). The high standard error (SE) values indicate significant variability in DCB populations observed at fortnightly intervals under field conditions. This variability contributed to the elevated standard error, highlighting the heterogeneity within the data collected.

### 3.3. Highest and Lowest Pest Population

**Figure 2:**
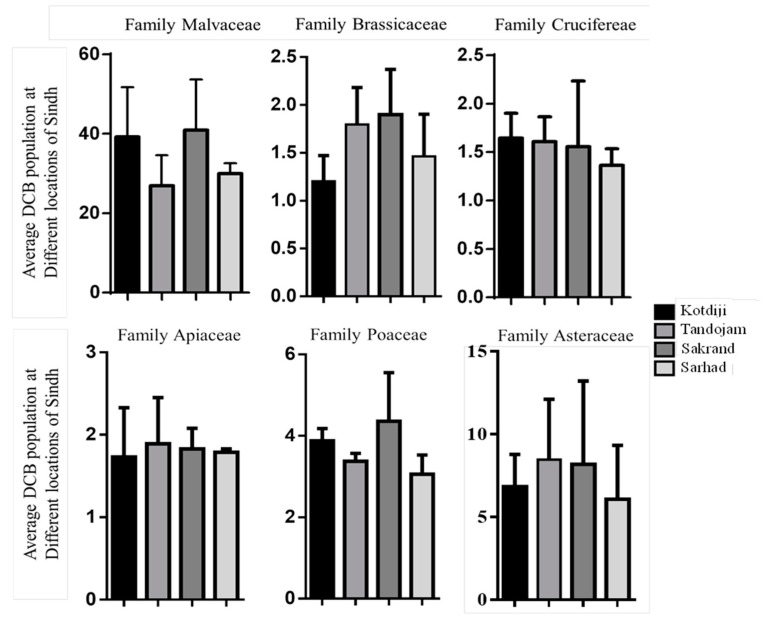
Total mean of pest population on plant families at various locations of Sindh.

The present outcomes (Figure 2) showed that the highest pest populations were recorded in Sakrand (40.92 DCB/shoot), Kotdiji (39.28 DCB/shoot), Sarhad (29.98 DCB/shoot), and Tandojam (26.95 DCB/shoot) on plants from the Malvaceae family. Conversely, the lowest pest numbers were recorded in Kotdiji (1.65 DCB/plant) and Tandojam (1.61 DCB/shoot) on plants from the Crucifereae family. Similarly, low pest populations were noted in Sakrand (1.90 DCB/shoot) and Tandojam (1.80 DCB/shoot) on plants from the Brassicaceae family.

Additionally, low pest populations were observed in Tandojam (1.89 DCB/shoot) and Sakrand (1.83 DCB/shoot) on plants from the Apiaceae family. Pest populations on plants from the Poaceae family were recorded in Sakrand (4.36 DCB/shoot) and Kotdiji (3.88 DCB/shoot). In the Asteraceae family, pest numbers were recorded in Tandojam (8.47 DCB/shoot) and Sakrand (8.19 DCB/shoot). An analysis of variance (ANOVA) was conducted to evaluate differences in pest populations across various plant families, revealing no statistically significant differences. The results for each family are as follows: Malvaceae (F = 0.49; Df = 3, 4; *p* = 0.71), Brassicaceae (F = 0.64; Df = 3, 7; *p* = 0.61), Cruciferae (F = 0.31; Df = 3, 7; *p* = 0.82), Apiaceae (F = 0.02; Df = 3, 4; *p* = 0.99), Poaceae (F = 0.74; Df = 3, 4; *p* = 0.58), and Asteraceae (F = 0.10; Df = 3, 4; *p* = 0.96). High standard error (SE) values point to pronounced fluctuations in DCB populations observed biweekly under field conditions, suggesting substantial variability within the dataset.

### 3.4. Different Plant Families

**Figure 3:**
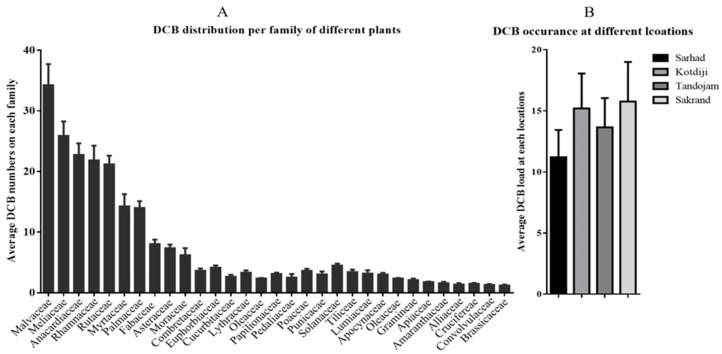
Distribution and occurrence of DCB across different plant families and locations. (**A**) Average DCB numbers observed across various plant families, with the highest values found in the Malvaceae, Megastigmaceae, and Anacardiaceae families. (**B**) Average DCB load recorded at four distinct locations (Sarhad, Kotdiji, Tandojam, and Sakrand), indicating varying DCB presence across these sites. Error bars represent standard deviations..

The current study (Figure 3) indicated that the pest population varied across different plant families. Some hosts exhibited high pest populations, while others had low pest populations.

The highest pest numbers were recorded in the Anacardiaceae family (43.95 ± 3.74), followed by Malvaceae (34.28 ± 8.90), Rutaceae (30.76 ± 9.16), Rhamnaceae (21.89 ± 2.39), Myrtaceae (14.31 ± 3.54), Palmaceae (14.03 ± 1.07), Fabaceae (9.38 ± 2.24), Meliaceae (9.25 ± 0.50), Asteraceae (7.40 ± 3.46), and Moraceae (6.21 ± 1.14). Conversely, lower pest numbers were noted in the Convolvulaceae family, (1.36 ± 0.09), Alliaceae (1.41 ± 0.19), Crucifereae (1.54 ± 0.13), Brassicaceae (1.59 ± 0.37), Amaranthaceae (1.63 ± 0.19), Apiaceae (1.81 ± 0.34), Gramineae (2.20 ± 0.30), and Oleaceae (2.39 ± 0.07).

### 3.5. Weather Factors

**Figure 4:**
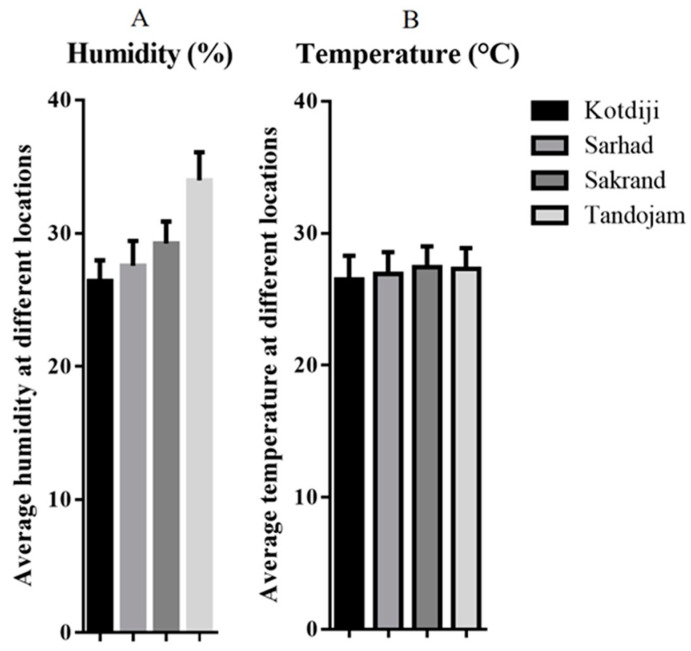
Overall average of weather factors in different research areas of Sindh in 2019. (**A**) Average relative humidity (%) at each location. (**B**) Average temperature (°C) recorded at each location. Locations include Kotdiji, Sarhad, Sakrand, and Tandojam. Error bars represent standard deviation.

The weather factors (Figure 4) in different agro-ecological zones of Sindh, such as temperature and humidity, were closely related. However, the mean temperature and relative humidity recorded in 2019 were as follows: Sarhad (26.93 °C, 27.56 RH), Kotdiji (26.5 °C, 26.40 RH), Tandojam (27.32 °C, 33.96 RH), and Sakrand (27.44 °C, 29.22 RH). While the temperatures were relatively close across the different locations, humidity levels varied more significantly.

The outcomes (Figure 4) indicated that high temperature was noted at Sakrand and Tandojam, with moderate temperature at Kotdiji and Sarhad. This study highlights the influence of weather factors, specifically temperature and relative humidity, in different agro-ecological zones of Sindh, an area where similar research has not been previously reported. Furthermore, all alternate host plants and plant families identified in this study are newly documented in Sindh, Pakistan. Similarly, the highest relative humidity was recorded in Tandojam and Sakrand, while lower humidity levels were observed in Kotdiji and Sarhad.

## 4. Discussion

*O. laetus* is a migratory, polyphagous, and significant insect pest of cotton. Previous research has identified multiple alternate host plants for this pest, with [21] identifying twenty-three alternate host plants, including Guava, Mango, Jaman, Eucalyptus, and Neem, which are highly preferred by *O. laetus* due to their moisture content. This aligns with the findings by [22], who noted a variety of alternate host plants that sustain the pest year-round. The availability of seeds from these plants at different times provides a con-tinuous food source for *Oxycarenus* spp. [14,24]. The current study presents new findings on the various host plants of the Dusky Cotton Bug across the diverse climatic conditions of Sindh. To date, no similar research has been conducted in this region.

The present study supports these findings and expands the list of preferred host plants to include Cotton, Lemon, Moringa [11,19], Abutilon, Phalsa, Sweet lemon, Bar-seem, Wheat, Ficus, Jasmine, and Date palm [20]. The pest also feeds on various vegetables and fruits such as corn, apples, grapes, peaches, avocado, pineapple, pomegranate, figs, and dates [25]. Correlations between the population of the Dusky Cotton Bug (DCB) and climatic factors, specifically temperature and relative humidity, in various agro-ecological zones of Sindh were examined in a previous study. This research demonstrated a negative correlation between the DCB population and weather factors under field conditions across different agro-ecological zones of Sindh. These findings have been published in previous research papers [9,23]. This broad host range underscores the pest’s adaptability and po-tential for significant agricultural impact. *O. laetus* is particularly notorious within the Malvaceae family, where it feeds on young petiole tissues. The study’s findings indicate a high pest population in families such as Malvaceae, Anacardiaceae, Rutaceae, Rham-naceae, Myrtaceae, and Palmaceae. This is consistent with the observations of [26], who reported similar pest distributions in Fabaceae, Solanaceae, Euphorbiaceae, Asteraceae, and other families. The presence of these host plants serves as a reservoir for the pest, es-pecially during periods when primary crops are unavailable, as noted by [31].

The peak pest population was observed from September to October across various climatic zones of Sindh, with the first arrival noted in early September at Kotdiji. The ideal conditions for pest multiplication were found to be temperatures between 27 and 34 °C and relative humidity levels between 21% and 28% [28]. These conditions align with previous reports that suggest a significant increase in *O. laetus* populations during the cooler months of October and November, coinciding with the breeding season on highly opened cotton bolls [24,32]. The geographical dispersal, abundance, and infestation patterns of *O. laetus* are closely linked to its feeding and reproductive capabilities on wide hosts and adaptability to various environmental conditions [29]. The proximity of alternate host plant species to major crops in the agro-ecological zones of Sindh facilitates the dispersion of Dusky Cotton Bugs to primary agricultural fields. Identifying these host plants is crucial for developing Integrated Pest Management strategies that can mitigate the impact of this pest on cotton and other crops. This study highlights the importance of the continuous monitoring and management of *O. laetus* populations, particularly in regions with diverse agro-ecological zones. By understanding the pest’s behavior, host preferences, and environmental conditions that favor its proliferation, effective control measures can be implemented to protect crops and ensure sustainable agricultural practices.

## 5. Conclusions

This study identified sixty-three alternate host plants, spanning thirty-one plant families, infected by DCB across different agro-ecological zones of Sindh. The survival of DCB is heavily dependent on the availability of these alternate hosts, which provide shelter during extreme weather conditions. The findings of this research study provide innovative insights into the various climatic zones of Sindh. They contribute to a better understanding of the emerging trends in pest infestation.

The research offers novel insights into the infestation patterns of DCB, highlighting the pest’s adaptability to various climatic zones of Sind. This understanding is crucial for devising effective preventive measures against this significant pest.

The diversity of host plants not only sustains the DCB but also suggests potential infestations in other plants, particularly during different seasonal and growth periods of medicinal plants, herbs, and shrubs. At the end of the summer season, the DCB shows a preference for breeding on mature cotton bolls, a period marked by moderate temperatures and relative humidity, which favor pest multiplication. During the winter months, from December to March, the pest hibernates on various alternate host plants due to the limited availability of food. To mitigate the impact of DCB, it is essential to evaluate and manage these host plants through Integrated Pest Management (IPM) programs. Such measures would significantly reduce the pest’s survival and proliferation, thereby protecting cotton crops and other susceptible plants. This study’s findings underscore the importance of continuous monitoring and adaptive pest control strategies to manage the threat posed by the Dusky Cotton Bug effectively.

## Figures and Tables

**Table 1:** List of alternate host plants of Dusky Cotton Bug in different agro-eco zones of Sindh, Pakistan.

S.N.	Scientific Name	Family	Status	Kotdiji	Tandojam	Sakrand	Sarhad	Mean ± S.E	Cat
1	*Psidium guajava* L.	Myrtaceae	T	21.15 ± 5.87	26.53 ± 3.71	15.15 ± 3.90	8.33 ± 2.37	17.79 ± 3.92	A
2	*Syzygium cumini*	Myrtaceae	T	9.46 ± 2.29	8.92 ± 2.19	7.08 ± 1.53	4.92 ± 1.17	7.595 ± 1.03	A
3	*Eucalyptus camaldulensis* Dehnh	Myrtaceae	T	29.75 ± 6.76	27.13 ± 5.65	18.54 ± 3.67	14.79 ± 3.26	22.55 ± 3.53	A
4	*Melaleuca leucadendron*	Myrtaceae	T	10.38461538	5.79 ± 1.49	12.23 ± 1.13	8.77 ± 1.03	9.29 ± 1.37	A
5	*Ficus bengalensis* L.	Moraceae	T	5.46 ± 1.21	9.23 ± 1.54	3.62 ± 0.87	3.29 ± 0.74	5.40 ± 1.36	A
6	*Ficus benjamina*	Moraceae	T	6.64 ± 1.95	5.79 ± 1.49	3.38 ± 0.56	1.92 ± 0.29	4.43 ± 1.08	A
7	*Ficus religiosa* L.	Moraceae	T	4.62 ± 0.90	9.62 ± 1.84	5.77 ± 1.03	2.54 ± 0.54	5.46 ± 1.49	A
8	*Morus alba*	Moraceae	T	13.79 ± 3.10	7.85 ± 0.91	11.46 ± 5.75	5.08 ± 0.87	9.54 ± 1.93	A
9	*Citrus sinensis* Osbeck	Rutaceae	T	42.07 ± 8.93	36.83 ± 6.58	45.17 ± 4.11	35.58 ± 3.88	39.91 ± 2.25	B
10	*Citrus aurantifolia*	Rutaceae	T	19.40 ± 4.95	20.58 ± 1.80	27.75 ± 3.28	18.67 ± 2.53	21.60 ± 2.09	A
11	*Dalbergia sissoo* Roxb.	Fabaceae	T	11.74 ± 3.26	9.53 ± 2.39	12.85 ± 1.02	8.85 ± 0.94	10.74 ± 0.93	A
12	*Acacia nilotica* L.	Fabaceae	T	16.14 ± 4.26	13.71 ± 3.58	14.86 ± 4.20	9.86 ± 2.71	13.64 ± 1.35	A
13	*Cassia fistula* Linn.	Fabaceae	T	6.29 ± 0.92	4.89 ± 0.93	6.22 ± 1.48	5.42 ± 0.74	5.72 ± 0.33	A
14	*Acacia sengal*	Fabaceae	T	20.79 ± 5.03	15.36 ± 2.74	11.08 ± 1.15	9.15 ± 0.99	14.10 ± 2.58	A
15	*Vigna radiata* L.	Fabaceae	PC	1.88 ± 0.30	2.77 ± 0.28	2.31 ± 0.35	3.85 ± 0.45	2.70 ± 0.42	A
16	*Trifolium alexandrinum*	Fabaceae	FC	1.75 ± 0.45	1.83 ± 0.56	1.58 ± 0.45	1.75 ± 0.53	1.73 ± 0.05	A
17	*Triticum aestivum* L.	Gramineae	CC	1.22 ± 0.22	2.56 ± 0.56	2.89 ± 0.73	1.67 ± 0.41	2.09 ± 0.39	A
18	*Zea mays* L.	Gramineae	CC	0.89 ± 0.26	1.33 ± 0.24	2.11 ± 0.35	1.33 ± 0.24	1.42 ± 0.25	A
19	*Sorghum bicolor* (L.) Moench	Gramineae	CC	2.22 ± 0.40	2.89 ± 0.51	1.78 ± 0.49	2.89 ± 0.89	2.45 ± 0.27	A
20	*Cenchrus americanus* (L.) Morrone	Gramineae	CC	2.67 ± 0.60	3.15 ± 0.46	2.62 ± 0.37	2.92 ± 0.49	2.84 ± 0.12	A
21	*Cynodon dactylon* Pres.	Gramineae	W	1.22 ± 0.22	2.46 ± 0.37	1.92 ± 0.29	2.38 ± 0.29	2.00 ± 0.29	A
22	*Capsicum annum*	Solanaceae	V	1.83 ± 0.44	2.08 ± 0.26	1.92 ± 0.47	1.62 ± 0.33	1.86 ± 0.10	A
23	*Solanum melongena* L.	Solanaceae	V	12.29 ± 1.31	14.76 ± 1.78	9.69 ± 1.32	11.35 ± 1.35	12.25 ± 1.46	A
24	*Solanum lycopersium* L.	Solanaceae	V	1.88 ± 0.30	4.38 ± 0.63	5.08 ± 0.82	5.88 ± 0.95	4.30 ± 0.86	A
25	*Solanum tuberosum*	Solanaceae	V	1.22 ± 0.22	2.54 ± 0.53	3.15 ± 0.54	2.46 ± 0.29	2.45 ± 0.25	A
26	*Nicotiana tabacum* L.	Solanaceae	TP	2.56 ± 0.71	2.38 ± 0.37	1.85 ± 0.27	2.08 ± 0.26	2.22 ± 0.16	A
27	*Momordica charantia*	Cucurbitaceae	V	2.89 ± 0.42	2.23 ± 0.36	2.85 ± 0.50	1.85 ± 0.22	2.45 ± 0.25	A
28	*Cucumis sativus* L.	Cucurbitaceae	SP	2.11 ± 0.63	2.38 ± 0.43	1.85 ± 0.22	1.62 ± 0.21	1.99 ± 0.17	A
29	*Praecitrullus fistulosus* (Stocks) Pangalo	Cucurbitaceae	V	3.78 ± 0.55	3.15 ± 0.88	2.77 ± 0.84	1.92 ± 0.36	2.91 ± 0.39	A
30	*Luffa cylindrica* Linn	Cucurbitaceae	V	1.33 ± 0.24	2.23 ± 0.43	1.77 ± 0.40	1.54 ± 0.33	1.72 ± 0.19	A
31	*Abelmoschus esculentus* L.	Malvaceae	V	51.75 ± 8.15	34.63 ± 3.51	53.71 ± 4.68	32.63 ± 3.83	43.18 ± 5.54	B
32	*Hibiscus* sp.	Malvaceae	S	26.80 ± 4.40	19.27 ± 3.14	28.13 ± 3.88	27.33 ± 2.99	25.38 ± 2.06	B
33	*Brassica campestris* L.	Brassicaceae	OC	1.44 ± 0.41	2.56 ± 0.41	2.78 ± 0.64	2.31 ± 0.54	2.27 ± 0.29	A
34	*Brassica napus* L.	Brassicaceae	OC	0.67 ± 0.17	1.33 ± 0.39	1.17 ± 0.42	0.83 ± 0.36	1.00 ± 0.15	A
35	*Brassica rapa* L.	Brassicaceae	V	1.50 ± 0.33	1.50 ± 0.58	1.75 ± 0.57	1.25 ± 0.43	1.50 ± 0.10	A
36	*Raphanus satvus*	Cruciferae	V	1.38 ± 0.38	1.83 ± 0.63	1.67 ± 0.44	1.42 ± 0.50	1.57 ± 0.11	A
37	*Lagenaria siceraria* (Molina) standl	Crucifereae	V	1.89 ± 0.20	1.33 ± 0.54	0.83 ± 0.36	1.17 ± 0.47	1.31 ± 0.22	A
38	*Brassica olerace*	Crucifereae	V	1.67 ± 0.24	1.67 ± 0.62	2.17 ± 0.82	1.50 ± 0.58	1.75 ± 0.14	A
39	*Foeniculum vulgare* Mill.	Apiaceae	MP	2.33 ± 0.44	2.45 ± 0.90	2.08 ± 0.84	1.75 ± 0.71	2.15 ± 0.16	A
40	*Daucus carota*	Apiaceae	V	1.13 ± 0.23	1.33 ± 0.02	1.58 ± 0.54	1.83 ± ± 0.73	1.47 ± 0.15	A
41	*Bambusa vulgaris*	Poaceae	T	3.57 ± 0.65	3.57 ± 0.61	3.17 ± 0.70	2.58 ± 0.99	3.22 ± 0.23	A
42	*Saccharum officinarum* L.	Poaceae	SC	4.18 ± 1.09	3.18 ± 0.80	5.55 ± 1.03	3.53 ± 0.49	4.11 ± 0.52	A
43	*Halianthus annuus* Linn.	Asteraceae	OSC	8.78 ± 2.74	12.11 ± 2.78	13.22 ± 3.99	9.33 ± 2.74	10.86 ± 1.07	A
44	*Cichorium intybus*	Asteraceae	FC	4.89 ± 0.93	4.83 ± 2.79	3.17 ± 0.95	2.83 ± 0.79	3.93 ± 0.54	A
45	*Grewia asiatica* L.	Tiliceae	FP	2.49 ± 0.39	4.13 ± 0.77	3.73 ± 0.51	3.53 ± 0.41	3.47 ± 0.35	A
46	*Phoenix dactylifera* L.	Palmaceae	T	13.54 ± 3.79	13.92 ± 1.99	16.92 ± 3.22	11.75 ± 1.75	14.03 ± 1.07	A
47	*Mangifera indica* L.	Anacardiaceae	T	51.65 ± 11.99	43.92 ± 8.63	46.42 ± 5.84	33.83 ± 4.64	43.95 ± 3.74	B
48	*Zizyphus mauritiana* Lamk	Rhamnaceae	T	22.87 ± 5.07	22.33 ± 5.18	26.92 ± 3.52	15.42 ± 3.67	21.89 ± 2.39	A
49	*Sesamum indicum* Linn.	Pedaliaceae	OSC	1.38 ± 0.38	3.58 ± 1.20	3.33 ± 0.81	1.92 ± 0.66	2.55 ± 0.54	A
50	*Allium cepa* L.	Alliaceae	V	0.88 ± 0.23	1.58 ± 0.45	1.75 ± 0.48	1.42 ± 0.45	1.41 ± 0.19	A
51	*Spinacia oleracea* L.	Amaranthaceae	Ve	1.17 ± 0.31	1.75 ± 0.53	2.08 ± 0.50	1.5 ± 0.50	1.63 ± 0.19	A
52	*Convolvulus arvensis*	Convolvulaceae	W	1.11 ± 0.11	1.5 ± 0.50	1.42 ± 0.45	1.42 ± 0.40	1.36 ± 0.09	A
53	*Sesbania sesban*	Papilionaceae	FC	1.56 ± 0.24	1.33 ± 0.44	2.55 ± 0.70	1.91 ± 0.36	1.84 ± 0.27	A
54	*Pongamia pinnata* L.	Papilionaceae	T	5.64 ± 1.39	4.33 ± 0.96	3.83 ± 0.97	4.17 ± 1.06	4.49 ± 0.40	A
55	*Azadirachta indica* A. Juss.	Meliaceae	T	8.67 ± 1.81	9.92 ± 1.42	10.25 ± 1.64	8.17 ± 1.61	9.25 ± 0.50	A
56	*Combretum indicum*	Combretaceae	S	4.43 ± 0.99	3.17 ± 0.97	4.08 ± 1.01	3.08 ± 0.66	3.69 ± 0.33	A
57	*Ricinus communis* L.	Euphorbiaceae	S	5.14 ± 1.02	4.17 ± 0.76	3.67 ± 0.91	3.83 ± 0.76	4.20 ± 0.33	A
58	*Lawsonia inermis* Linn	Lythraceae	S	4.57 ± 0.84	3.67 ± 0.98	3.33 ± 0.69	2.42 ± 0.54	3.50 ± 0.44	A
59	*Calotropis procera* R. Br.	Apocynaceae	S	3.46 ± 0.78	2.58 ± 0.45	3.42 ± 0.62	2.83 ± 0.63	3.07 ± 0.22	A
60	*Jasminum officinale* L.	Oleaceae	OP	2.42 ± 0.50	2.56 ± 0.50	2.33 ± 0.54	2.25 ± 0.61	2.39 ± 0.07	A
61	*Ocimum basilicum*	Lumiaceae	OP	2.50 ± 0.58	4.75 ± 0.92	2.58 ± 0.50	3.00 ± 0.48	3.21 ± 0.53	A
62	*Rosa indica*	Rosaceae	OP	2.25 ± 0.64	1.78 ± 0.32	2.33 ± 0.44	2.42 ± 0.45	2.20 ± 0.14	A
63	*Punica granatum* Linn	Punicacae	T	2.57 ± 0.60	4.07 ± 0.74	3.57 ± 0.65	2.17 ± 0.32	3.09 ± 0.44	A

Legends: T = Tree; PC = Pulse crop; TP = Tobacco plant; OP = Ornamental Plant; S = Shrub; FC = Fodder crops; V = Vegetable OSC = Oilseed crop; CC = Cereal crop; W = Weed; SP = Salad plant; MP = Medicinal plant; SC = Sugar crop; FP = Fruit plant.

## Data Availability

The data presented in this study are available on request from the corresponding author.
